# Supplementary material for: RNA modifications and Prp24 coordinate Lsm2–8 binding dynamics during S. cerevisiae U6 snRNP assembly
Source: J Biol Chem. 2025 Apr 10;301(5):108497. doi: 10.1016/j.jbc.2025.108497 (PMC12136795; doi:10.1016/j.jbc.2025.108497)
Supplement: Supplemental data [file mmc1.pdf]

RNA Modifications and Prp24 Coordinate Lsm2-8 Binding Dynamics during *S. cerevisiae* U6 snRNP Assembly

Ye Liu[1], Yuichiro Nomura[1], Samuel E. Butcher[1] and Aaron A. Hoskins[1,2,*]

[1] Department of Biochemistry, University of Wisconsin-Madison, Madison, WI 53706

[2] Department of Chemistry, University of Wisconsin-Madison, Madison, WI 53706

* To whom correspondence should be addressed. Tel: +1 608 890 3101; Fax: +1 608 265 4693; Email: ahoskins@wisc.edu

This supplementary file contains:

- Supplementary Figures S1-S4
- Supplementary Tables S1-S4

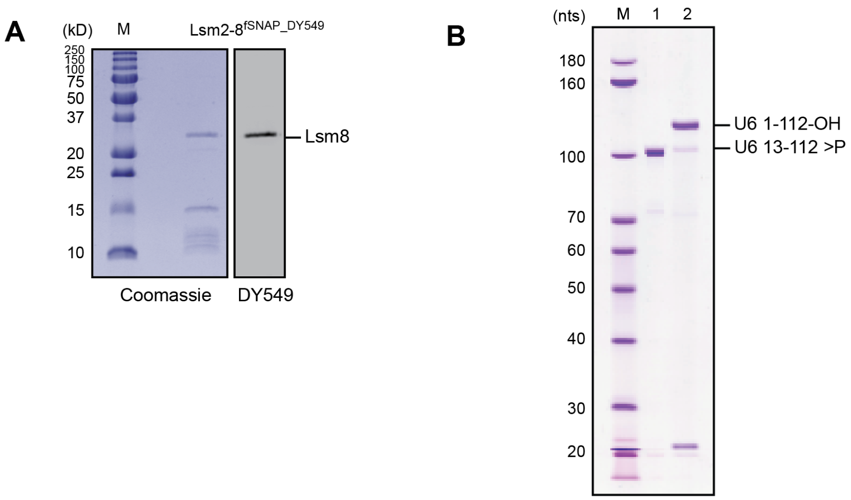

Supplementary Fig S1. Denaturing PAGE analysis of fluorophore-labelled reagents. (A) SDS-PAGE analysis of the purified Lsm2-8^{fSNAP} labeled with DY549. **(B)** Denaturing urea PAGE gel showing the U6 13-112 transcripts (lane 1) and final U6 1-112 ligation product (lane 2).

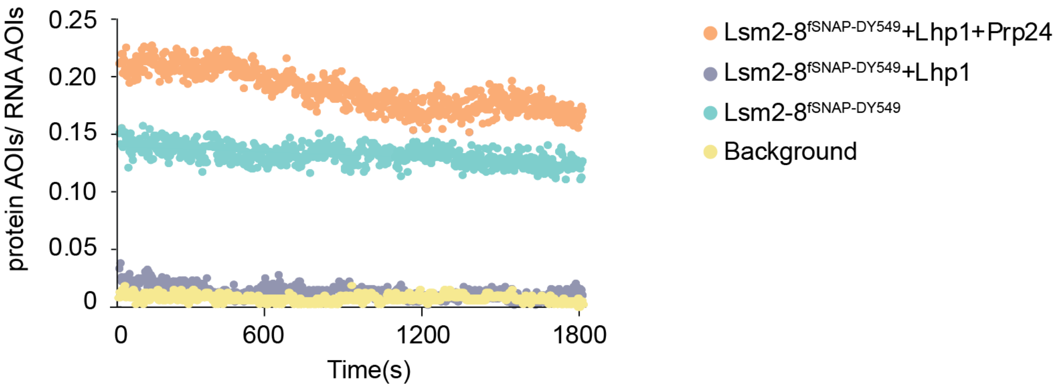

Supplementary Figure S2. Surface accumulation of fluorescent Lsm2-8^{fSNAP} molecules. Spot accumulation trajectories (normalized to the number of immobilized RNAs or blank AOIs (background)) are compared for the Lsm2-8 under conditions in which the U6 RNA 3' end is freely accessible (teal, orange) or bound by Lhp1 (purple).

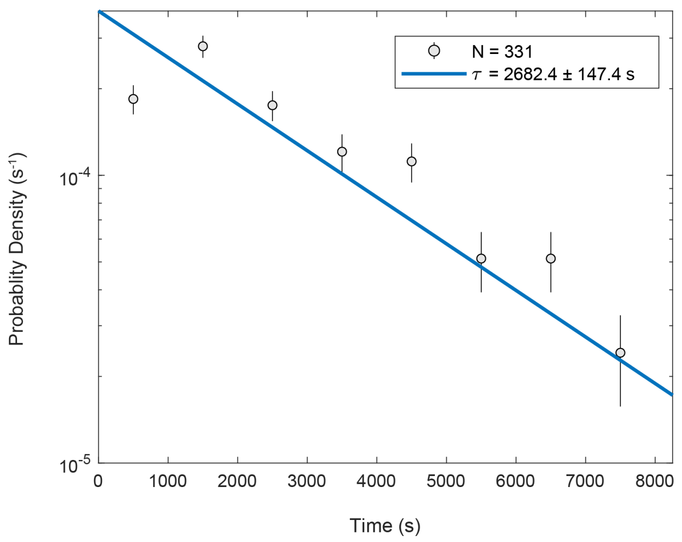

Supplementary Figure S3. Fluorescent lifetime of DY549-labelled fSNAP protein. Biotinylated fSNAP protein labeled with DY549 was immobilized on a glass slide, and imaged under the same conditions as the CoSMoS assays used for monitoring Lsm2-8 dynamics. Points represent the centers of histogram bins and errors bars represent the standard deviation assuming a binomial distribution where $\sigma = \sqrt{NP(1-P)}$ and N and P represent the population size and probability of an event being within the given bin, respectively. The lifetime distribution was fit to a single exponential equation, yielding a time constant (τ) of 2682.4±147.5s.

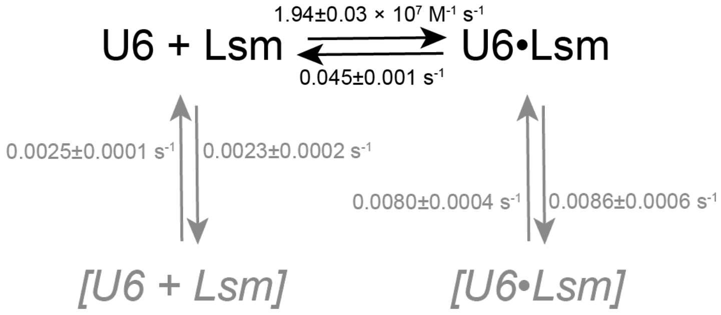

Supplementary Figure S4. A kinetic model for the interaction between 3' end modified U6 RNA and the Lsm2-8 complex. The 3' end modified U6 snRNA (U6; 1-112-P) can reversibly bind with the Lsm2-8 complex (Lsm). At slower rates, the substrates and products can occasionally transition to states from which the RNA and protein can neither directly associate or dissociate (grey arrows and italicized complexes).

Supplementary Table 1. Fitted binding affinity parameters from fluorescence polarization assays.

RNA	Lsm2-8^{WT}			Lsm2-8^{fSNAP}		
**U6 3' tail**	**K_D (nM)**	**Hill coefficient**	**R^2**	**K_d (nM)**	**Hill coefficient**	**R^2**
U6 95-113-OH	6.75±1.15	1.18±0.24	0.98	6.70±1.10	1.12±0.22	0.98
U6 95-112-OH	84.64±16.82	1.26±0.26	0.97	113.80±16.80	1.35±0.24	0.98
U6 95-112-P	10.34±4.10	1.11±0.45	0.89	12.83±3.87	0.88±0.23	0.95

Supplementary Table 2. Fitted unbound time kinetic parameters from dwell time analysis.

Tagged RNA	Other protein added	Amplitude 1	T1 (s)	Amplitude 2	T2 (s)	Number of Events Fit
U6 1-113-OH	-	0.50±0.03	85.60±10.40	0.50±0.03	559.80±112.30	997
U6 1-112-P	-	0.81±0.03	32.40±6.40	0.19±0.03	370.75±28.45	2401
U6 1-112-OH	-	0.72±0.06	166.05±82.95	0.28±0.06	333.05±52.95	2657
U6 1-113+5U-OH	-	0.67±0.01	117.70±3.25	0.33±0.01	403.50±29.85	1113
U6 1-113-OH	Prp24	0.60±0.04	33.35±16.65	0.40±0.04	354.15±51.25	1142
U6 1-112-P	Prp24	0.65±0.03	42.60±12.00	0.36±0.03	404.45±146.45	2303
U6 1-112-OH	Prp24	0.83±0.02	74.10±8.00	0.17±0.02	280.10±69.9	1461
U6 1-113+5U-OH	Prp24	0.75±0.03	120.40±14.90	0.25±0.03	382.20±24.10	2827
U6 1-112-P	Prp24$^{\Delta SNFFL}$	0.82±0.07	112.80±8.30	0.18±0.07	442.90±38.70	1668
U6 1-113+5U-OH	Prp24$^{\Delta SNFFL}$	0.70±0.03	126.80±9.10	0.30±0.03	399.30±4.20	1991

Supplementary Table 3. Fitted bound time kinetic parameters from dwell time analysis.

Tagged RNA	Other protein added	Amplitude 1	T1 (s)	Amplitude 2 (s)	T2	Number of Events Fit
U6 1-113-OH	-	0.80±0.10	20.85±8.75	0.20±0.10	183.10±8.60	911
U6 1-112-P	-	0.79±0.07	30.90±14.90	0.21±0.07	226.80±54.30	2402
U6 1-112-OH	-	0.99±0.01	12.40±3.60	0.01±0.01	304.60±199.50	2454
U6 1-113+5U -OH	-	0.88±0.02	20.90±1.80	0.12±0.02	194.60±80.10	1078
U6 1-113-OH	Prp24	0.45±0.03	29.40±6.10	0.55±0.03	290.30±58.50	1121
U6 1-112-P	Prp24	0.69±0.06	32.4±4.6	0.31±0.06	295.40±160.60	2337
U6 1-112-OH	Prp24	0.88±0.07	19.70±1.30	0.12±0.07	282.20±48.90	1406
U6 1-113+5U -OH	Prp24	0.90±0.03	24.80±1.00	0.10±0.03	255.90±5.50	2642
U6 1-112-P	Prp24$^{\Delta SNFFL}$	0.85±0.01	19.70±0.70	0.15±0.01	143.25±24.45	1519
U6 1-113+5U -OH	Prp24$^{\Delta SNFFL}$	0.89±0.01	22.60±1.70	0.21±0.01	267.10±72.50	1802

Supplementary Table 4. Synthetic Oligonucleotide Sequences.

Oligo #	ID	Sequence (5'-3')	Length (nts)
1	FAM_U6_95-113-OH	5' - /56-FAM/ rArGrArGrArUrUrUrArUrU rUrCrGrUrUrUrUrU - 3'	19
2	FAM_U6_95-112-P	5' - /56-FAM/ rArGrArGrArUrUrUrArUrU rUrCrGrUrUrUrU/3Phos/ - 3'	18
3	FAM_U6_95-112-OH	5' - /56-FAM/ rArGrArGrArUrUrUrArUrU rUrCrGrUrUrUrU - 3'	18
4	Bio_Cy5_U6_1-12	5' - /5Biosg/ rGrUrUrCrGrC/iCy5/rGrAr ArGrUrA- 3'	12
5	DNA 12-13 bridge	5' - GTCCACGAAGGGTTAC TTCGCGAAC - 3'	25
6	U6 95-112-P	5' - /5Phos/rArGrArGrArUrUr UrArUrUrUrCrGrUrUrUrU /3Phos/ - 3'	18
7	DNA 94-95 bridge	5' – GAAATAAATCTCTTTGT AAAACGGTTC - 3'	27
